# Gemcitabine based combination chemotherapy in advanced pancreatic cancer-indirect comparison

**DOI:** 10.1186/1471-2407-8-192

**Published:** 2008-07-08

**Authors:** Asma Sultana, Paula Ghaneh, David Cunningham, Naureen Starling, John P Neoptolemos, Catrin Tudur Smith

**Affiliations:** 1 CRUK Liverpool Cancer Trials Unit, Cancer Research Centre, 200 London Road, Liverpool, L3 9TA, UK; 2 Department of Medicine, Royal Marsden Hospital, Downs Road, Sutton, Surrey SM2 5PT, UK; 3 Centre for Medical Statistics and Health Evaluation, University of Liverpool, Shelley's Cottage, Brownlow Street, Liverpool, L69 3GS, UK

## Abstract

**Background:**

Recent meta-analyses have found a survival advantage with gemcitabine based combinations over single agent gemcitabine in patients with advanced pancreatic cancer. There is paucity of evidence in the form of direct head-to-head randomised controlled trials to determine which combinations are to be preferred.

**Method:**

Using the adjusted indirect comparison method proposed by Bucher et al, we have assessed randomised controlled trials of four gemcitabine based combinations namely gemcitabine plus a platinum compound or 5-fluorouracil or irinotecan or capecitabine.

**Results:**

No particular combination was significantly superior to another, but the indirect evidence suggests some important trends.

**Conclusion:**

The strongest trends on indirect comparison are towards favouring gemcitabine plus capecitabine or gemcitabine plus a platinum compound over gemcitabine plus irinotecan, and to a lesser degree, over gemcitabine plus 5-fluorouracil.

## Background

We have previously reported a systematic review and meta-analysis of 19 studies evaluating gemcitabine based combination chemotherapy compared to gemcitabine alone [1] in patients with locally advanced and metastatic pancreatic cancer. Overall survival was significantly better for gemcitabine based combination chemotherapy (14 trials 4060 patients HR 0.91; 95% CI 0.85 to 0.97) compared to single agent gemcitabine. A subgroup analysis was performed, dividing the selected studies into four categories defined by the addition to gemcitabine of platinum agents or 5-fluorouracil (5FU) or irinotecan or capecitabine (Table 1). The subgroup analysis found evidence to suggest a survival advantage for gemcitabine combined with either a platinum agent (HR 0.85; 95% CI 0.74 to 0.96) or capecitabine (HR 0.83; 95% CI 0.72 to 0.96), and insufficient evidence to support combinations of gemcitabine with either 5FU (HR 0.98; 95% CI 0.86 to 1.11) or irinotecan (HR 1.01; 95% CI 0.84 to 1.22). These analyses provide estimates of the survival advantage for each combination compared to single agent gemcitabine but do not provide estimates of the survival advantage for each combination compared against another. To date, there is only one phase II randomised controlled trial [2,3] which directly compared different gemcitabine combinations in a head-to-head comparison. This was a small study that directly compared only two gemcitabine based combination chemotherapy regimens (gemcitabine plus capecitabine versus gemcitabine plus oxaliplatin). In view of the paucity of data directly comparing alternative gemcitabine based combinations, we have attempted to answer, for the first time, as to which combinations of gemcitabine show more promise, an important clinical question which no previous meta-analyses have addressed.

**Table 1:** List of included studies utilised in indirect comparison of gemcitabine based combination chemotherapy

**Comparison**	**Trial**	**Group (number randomised)**
**Gemcitabine versus gemcitabine plus capecitabine**	Cunningham 2005 (interim analyses) [12]	Gemcitabine (n = 266) Gemcitabine combination (n = 267)
	Hermann 2005 [13,14] (analyses based on data from abstract published in 2005, plus extra data provided by trialist)	Gemcitabine (n = 159) Gemcitabine combination (n = 160)
	Scheithauer 2003 [15]	Gemcitabine (n = 42) Gemcitabine combination (n = 41)
**Gemcitabine versus gemcitabine plus 5FU**	Berlin 2002 [8]	Gemcitabine (n = 162) Gemcitabine combination (n = 160)
	Di Costanzo 2005 [10]	Gemcitabine (n = 48) Gemcitabine combination (n = 43)
	Reiss 2005 [9]	Gemcitabine (n = 236) Gemcitabine combination (n = 230)
**Gemcitabine versus gemcitabine plus platinum compound**	Heinemann 2006 [16]	Gemcitabine (n = 99) Gemcitabine combination (n = 96)
	Louvet 2005 [17]	Gemcitabine (n = 163) Gemcitabine combination (n = 163)
	Poplin 2006 [18]	Gemcitabine (n = 280) Gemcitabine combination (n = 276)
**Gemcitabine versus gemcitabine plus irinotecan**	Rocha Lima 2004 [19]	Gemcitabine (n = 180) Gemcitabine combination (n = 180)
	Stathopoulos 2005 [20,21] (analyses based on data from abstract published in 2005, plus extra data provided by trialist)	Gemcitabine (n = 69) Gemcitabine combination (n = 57)

## Methods

We searched for direct comparisons of gemcitabine combinations, as well as used adjusted indirect comparisons to evaluate the treatment effect across studies [3,4] although this was not specified a priori. Illustration of how the indirect comparison was obtained is given in the following example. Suppose an intervention A was compared against another intervention C in a randomised controlled trial or meta-analysis, and likewise another study (or meta-analysis) compared intervention B with intervention C. Adjusted indirect comparison of treatments A versus B was obtained as follows:

(1) The log hazard ratio of the adjusted indirect comparison for intervention A versus B was calculated using the following formula:

log HR_AB _= log HR_AC_-log HR_BC_

where log HR_AC _was the log hazard ratio for the direct comparison of intervention A versus C and log HR_BC _was the log hazard ratio for the direct comparison of intervention B versus C.

(2) The standard error for the log hazard ratio was obtained using the calculation:

$$\text{SE }(\log{\text{HR}}_{\text{AB}})=\sqrt{\text{SE}{\left(\log{\text{HR}}_{\text{AC}}\right)}^{2}+\text{SE}{\left(\log{\text{HR}}_{\text{BC}}\right)}^{2}}$$

where SE(log HR_AC_) was the standard error of the log hazard ratio for the direct comparison of intervention A versus C and SE(log HR_BC_) was the standard error of the log hazard ratio for the direct comparison of intervention B versus C.

The assumption of exchangeable treatment effects (treatment effect observed in trials comparing A versus C is assumed to be the treatment effect that would have been observed in those trials comparing B versus C if treatment A had been included in those trials and vice versa) across comparisons was evaluated by assessing heterogeneity across trials within each comparison and assessing comparability (methodological and clinical characteristics) of all trials contributing to the indirect comparison.

## Results

Adjusted indirect comparisons were computed for the following comparisons:

1. Gemcitabine plus a platinum agent versus gemcitabine plus 5FU (GemPlat versus Gem5FU)

2. Gemcitabine plus a platinum agent versus gemcitabine plus capecitabine (GemPlat versus GemCap)

3. Gemcitabine plus a platinum agent versus gemcitabine plus irinotecan (GemPlat versus GemIrino)

4. Gemcitabine plus 5FU agent versus gemcitabine plus capecitabine (Gem5FU versus GemCap)

5. Gemcitabine plus 5FU agent versus gemcitabine plus irinotecan (Gem5FU versus GemIrino).

6. Gemcitabine plus capecitabine versus gemcitabine plus irinotecan (GemCap versus GemIrino)

**Figure 1:**
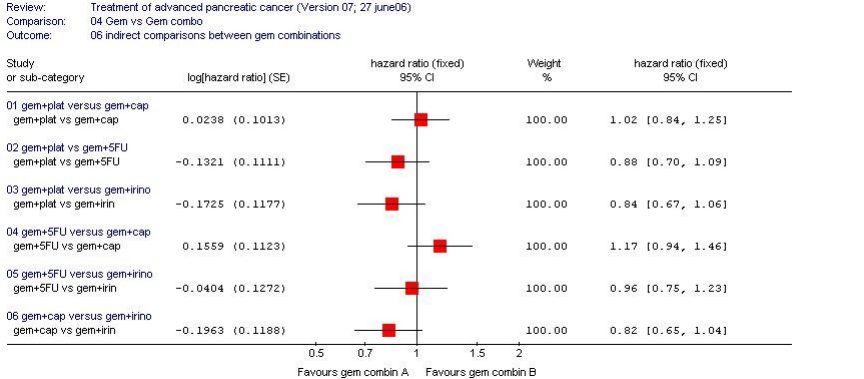
Indirect comparison between different gemcitabine-based combination chemotherapy regimens.

We did not find any one combination to be significantly superior to others (Fig 1), but the indirect evidence suggests some important trends, of which the strongest are towards favouring gemcitabine plus capecitabine or gemcitabine plus a platinum compound over gemcitabine plus irinotecan (GemCap versus GemIrino HR 0.82, 95% CI 0.65 to 1.04; GemPlat versus GemIrino HR 0.84, 95% CI 0.67 to 1.06). Some advantage, to a lesser degree, was suggested for these two combinations over gemcitabine plus 5FU (Gem5FU versus GemCap HR 1.17, 95% CI 0.94 to 1.46; GemPlat versus Gem5FU HR 0.88, 95% CI 0.70 to 1.09). There was no evidence for heterogeneity within the direct comparisons and no obvious inconsistency with the assumption of exchangeable treatment effects.

Overall survival data was extracted from the single phase II randomised trial to allow estimation of a HR (95% CI) [5] for the direct comparison between GemOx and GemCap. There was no significant difference in overall survival between the two combinations (HR 0.81, 95% CI 0.56 to 1.18) on direct head-to-head comparison [2] as well as on computing this combining the results of both the direct and indirect comparisons (HR 0.94, 95% CI 0.79 to 1.12) using random effects analysis.

## Discussion

In a situation wherein two drugs A and B have, in randomised controlled trials, shown to be effective in comparison to a placebo or common standard, but direct comparison between A and B is not available, indirect comparison can be used [4]. The use of a simple indirect comparison has the limitation that the difference detected may not be a true difference, but instead be attributable to variations in patient characteristics and other prognostic factors in the different trials. An adjusted indirect comparison method was proposed by Bucher et al, and this method maintains the randomisation of the originally assigned patients while calculating the magnitude of the treatment effect.

Song et al assessed the comparability of indirect with the direct head to head comparison in the setting of clinical trials dealing with antimicrobial prophylaxis in colorectal cancer, and later using a sample of 44 comparisons from 28 systematic reviews [3,6]. Compared with direct estimates, the adjusted indirect estimates were less likely to be statistically significant. Adjusted indirect comparisons usually but not always agree with the results of head to head randomised trials. When there is no or insufficient direct evidence from randomised trials, the adjusted indirect comparison can provide useful or supplementary information on the relative efficacy of competing interventions. The results of adjusted indirect comparisons should be interpreted with caution however and the internal and external validity of the trials involved examined carefully, to investigate potential causes of discrepancy.

This adjusted indirect comparison of gemcitabine-based combination chemotherapy is very important clinically as there is paucity of evidence comparing these combinations and this is the first time hazard ratios have ever been calculated for these pairwise comparisons. The only randomised trial comparing gemcitabine combinations was a phase II multicentre study that compared capecitabine plus oxaliplatin(CapOx) versus capecitabine plus gemcitabine (CapGem) versus gemcitabine plus oxaliplatin (mGemOx) [2]. There was no significant difference in the primary end point of progression free survival [median progression free survival time (p = 0.56) and progression free survival rates (p = 0.67)] and overall survival (GemCap versus GemOx HR 0.81; 95% CI 0.56–1.18). Grade 3/4 haematological toxicities were seen more often in the gemcitabine containing arms.

Although indirect evidence may not be as reliable as evidence from a randomised head to head comparison, these analyses show some interesting trends that could be used to direct future research priorities. The assumption of exchangeable treatment effects would seem reasonable for these comparisons which add strength to the clinical interpretation and conclusions. In particular, trends suggest that gemcitabine plus irinotecan may be the least effective of the combinations examined. The lack of significant differences on indirect comparison is probably due to the already highlighted observation that this method tends to yield results that are less statistically significant than in a direct comparison [6]. Indeed, it can be shown that one directly randomised trial is as precise as an indirect comparison based on four randomised trials of the same size.

A note-worthy observation on indirect comparison was that overall survival with gemcitabine combined with the fluoropyrimidine 5FU was inferior (though not statistically significant) to gemcitabine plus another fluoropyrimidine capecitabine (HR 1.17). A likely explanation is that capecitabine, an oral prodrug of 5FU, has the advantage of an element of tumour targeting, leading to enhanced selectivity and better tolerability [7]. The higher levels of thymidine phorphorylase (the final requisite enzyme for conversion of capecitabine to 5FU) observed in tumours compared to normal tissue may account for the improved targeting. Another possibility is the mode of delivery of 5FU versus capecitabine. The 5FU trials have involved bolus 5FU schedule [8] or 24 hour infusion [9], with the exception of one trial where 5FU was given by continuous infusion [10]. In contrast, the administration of capecitabine is more analogous to the delivery of 5FU by continuous protracted venous infusion, with the added ease of oral administration.

In the light of level I evidence demonstrating that gemcitabine based combinations have a modest survival advantage over single agent gemcitabine, the current study indicates which combinations may be more efficacious. The findings of our original meta-analyses, as well as the trends observed on our adjusted indirect comparisons support the use of gemcitabine in combination with either capecitabine or a platinum compound in clinical practice. Future randomised controlled trials will now likely to be centred on the exploitation of novel targets or biology (such as Telovac) [11] in this chemo-resistant cancer, probably on a cytotoxic backbone of a gemcitabine combination.

## Conclusion

Adjusted indirect comparison of randomised controlled trials examining gemcitabine in combination with capecitabine, platinum based compounds, 5FU and irinotecan reveal trends towards favouring gemcitabine plus capecitabine or gemcitabine plus a platinum compound over gemcitabine plus irinotecan and to a lesser degree, over gemcitabine plus 5-fluorouracil. Future trials will now likely to be centred on the exploitation of novel targets or biology, probably on a cytotoxic backbone of a gemcitabine combination.

## Abbreviations

CI: Confidence interval; GemCap: Gemcitabine plus capecitabine; GemIrino: Gemcitabine plus irinotecan; GemPlat: Gemcitabine plus platinum compound; HR: Hazard ratio.

## Competing interests

The authors declare that they have no competing interests.

## Authors' contributions

AS, CTS, JPN and PG were involved in the study design, data collection and analysis and manuscript write-up. DC and NS were involved in drafting the manuscript. All authors have read and approved the final manuscript.

## Pre-publication history

The pre-publication history for this paper can be accessed here:



## References

[B1] Sultana A, Tudur Smith C, Cunningham D, Starling N, Neoptolemos J, Ghaneh P (2007). Meta-Analyses of Chemotherapy for Locally Advanced and Metastatic Pancreatic Cancer. J Clin Oncol.

[B2] Boeck S, Hoehler T, Seipelt G, Mahlberg R, Wein A, Hochhaus A, Boeck H, Schmid B, Kettner E, Stauch M, Lordick F, Ko Y, Geissler M, Schoppmeyer K, Kojouharoff G, Golf A, Neugebauer S, Heinemann V (2008). Capecitabine plus oxaliplatin (CapOx) versus capecitabine plus gemcitabine (CapGem) versus gemcitabine plus oxaliplatin (mGemOx): final results of a multicentre randomised phase II trial in advanced pancreatic cancer. Ann Oncol.

[B3] Song F, Glenny A-M, Altman D (2000). Indirect comparison in evaluating relative efficacy illustrated by antimicrobial prophylaxis in colorectal surgery. Control Clin Trials.

[B4] Bucher H, Guyatt G, Griffith L, Walter S (1997). The results of direct and indirect treatment comparisons in meta-analysis of randomised controlled trials. J Clin Epidemiol.

[B5] Parmar M, Torri V, Stewart L (1998). Extracting summary statistics to perform meta-analyses of the published literature for survival endpoints. Statistics in Medicine.

[B6] Song F, Altman D, Glenny A-N, Deeks J (2003). Validity of indirect comparison for estimating efficacy of competing interventions: emperical evidence from published meta-analyses. BMJ.

[B7] Smith DB, Neoptolemos JP (2006). Capecitabine in carcinoma of the pancreas. Expert Opin Pharmacother.

[B8] Berlin JD, Catalano P, Thomas JP, Kugler JW, Haller DG, Benson AB (2002). Phase III study of gemcitabine in combination with fluorouracil versus gemcitabine alone in patients with advanced pancreatic carcinoma: Eastern Cooperative Oncology Group Trial E2297. J Clin Oncol.

[B9] Reiss H, Helm A, Niedergethmann M, Schmidt-Wolf I, Moik M, Hammer C, Zippel K, Weigang-Kohler K, Stauch M, Oettle H (2005). A randomised, prospective, multicentre, phase III trial of gemcitabine, 5-Fluorouracil, folinic acid versus gemcitabine alone in patients with advanced pancreatic cancer. ASCO Annual Meeting: 2005.

[B10] Di Costanzo F, Carlini P, Doni L, Massidda B, Mattioli R, Iop A, Barletta E, Moscetti L, Recchia F, Tralongo P, Gasperoni S (2005). Gemcitabine with or without continuous infusion 5-FU in advanced pancreatic cancer: a randomised phase II trial of the Italian oncology group for clinical research (GOIRC). Br J Cancer.

[B11] UK Clinical Research Network (2007). TeloVac: A prospective, phase III, controlled, multicentre, randomised clinical trial comparing combination Gemcitabine and Capecitabine therapy with concurrent and sequential chemoimmunotherapy using a telomerase vaccine in locally advanced and metastatic pancreatic cancer. http://pfsearch.ukcrn.org.uk/StudyDetail.aspx?TopicID=1&StudyID=2210.

[B12] Cunningham D, Chau I, Stocken D, Davies C, Dunn J, Valle J, Smith D, Steward W, Harper P, Neoptolemos J (2005). Phase III randomised comparison of gemcitabine versus gemcitabine plus capecitabine in patients with advanced pancreatic cancer. Eur J Can.

[B13] Hermann R, Bodoky G, Ruhstaller T, Glimelius B, Saletti P, Bajetta E, Schueller J, Bernhard J, Dietrich D, Scheithauer W (2005). Gemcitabine plus capecitabine versus gemcitabine alone in locally advanced or metastatic pancreatic cancer. A randomised phase III study of the Swiss Group for Clinical Cancer Research (SAKK) and the Central European Cooperative Oncology Group (CECOG). 2005 ASCO Annual Meeting: 2005; Orlando, USA.

[B14] Herrmann R, Bodoky G, Ruhstaller T, Glimelius B, Bajetta E, Schueller J, Saletti P, Bauer J, Figer A, Pestalozzi B, Kohne C, Mingrone W, Stemmer S, Tamas K, Kornek GV, Koeberle D, Cina S, Bernhard J, Dietrich D, Scheithauer W, Swiss Group for Clinical Cancer Research, Central European Cooperative Oncology Group (2007). Gemcitabine plus capecitabine compared with gemcitabine alone in advanced pancreatic cancer: a randomised, multicenter, phase III trial of the Swiss Group for Clinical Cancer Research and the Central European Cooperative Oncology Group. J Clin Oncol.

[B15] Scheithauer W, Schull B, Ulrich-Pur H, Schmid K, Raderer M, Haider K, Kwasny W, Depisch D, Schneeweiss B, Lang F, Kornek GV (2003). Biweekly high-dose gemcitabine alone or in combination with capecitabine in patients with metastastic pancreatic adenocarcinoma: a randomised phase II trial. Ann Oncol.

[B16] Heinemann V, Quietzsch D, Gieseler F, Gonnermann M, Schonekas H, Rost A, Neuhaus H, Haag C, Clemens M, Heinrich B, Vehling-Kaiser U, Fuchs M, Fleckenstein D, Geisierich W, Uthgennant D, Einsele H, Holstege A, Hinke A, Schalhorn A, Wilkowski R (2006). Randomised phase III trial of gemcitabine plus cisplatin compared with gemcitabine alone in advanced pancreatic cancer. J Clin Oncol.

[B17] Louvet C, Labianca R, Hammel P, Lledo G, Zampino M, Andre T, Zaniboni A, Ducreux M, Aitini E, Taieb J, Faroux R, Lepere C, de Gramont A (2005). Gemcitabine in combination with oxaliplatin compared with gemcitabine alone in locally advanced or metastatic pancreatic cancer: Results of a GERCOR and GISCAD phase III trial. J Clin Oncol.

[B18] Poplin E, Levy D, Berlin J, Rothenberg M, Cella D, Mitchell E, Alberts S, Benson A (2006). Phase III trial of gemcitabine (30-minute infusion) versus gemcitabine (fixed-dose rate infusion) versus gemcitabine + oxaliplatin in patients with advanced pancreatic cancer (E6201). J Clin Oncol.

[B19] Rocha Lima C, Green M, Rotche R, Miller W, Jeffrey G, Cisar L, Morganti A, Orlando N, Gruia G, Miller L (2004). Irinotecan plus gemcitabine results in no survival advantage compared with gemcitabine monotherapy in patients with locally advanced or metastatic pancreatic cancer despite increased tumor response rate. J Clin Oncol.

[B20] Stathopoulos G, Aravantinos G, Syrigos K, Kalbakis K, Karvounis N, Papakotoulas P, Boukovinas J, Potamianou A, Polyzos A, Christophillakis C, Georgoulias V (2005). A randomised phase III study of irinotecan/gemcitabine combination versus gemcitabine in patients with advanced/metastatic pancreatic cancer. 2005 ASCO Annual Meeting: 2005; Orlando, USA.

[B21] Stathopoulos G, Syrigos K, Aravantinos G, Polyzos A, Papakotoulas P, Fountzilas P, Potamianou A, Ziras N, Boukovinas J, Varthalitis J, Androulakis N, Kotsakis A, Samonis G, Georgoulias V (2006). A multicentre phase III trial comparing irinotecan-gemcitabine with gemcitabine monotherapy as first-line treatment in patients with locally advanced or metastatic pancreatic cancer. Br J Cancer.

